# Polylactide Modified with ZnO and Raspberry Leaf Extract as Active Food Packaging

**DOI:** 10.3390/ijms27094002

**Published:** 2026-04-29

**Authors:** Magdalena Zdanowicz, Małgorzata Mizielińska, Wojciech Jankowski

**Affiliations:** Center of Bioimmobilisation and Innovative Packaging Materials, Faculty of Food Sciences and Fisheries, West Pomeranian University of Technology in Szczecin, Janickiego 35, 71-270 Szczecin, Poland; mmizielinska@zut.edu.pl (M.M.); jw42757@zut.edu.pl (W.J.)

**Keywords:** active packaging, antiviral, biocomposites, gel permeation chromatography, nanoparticles, polylactide, raspberry leaf, waste management

## Abstract

The aim of the study was to modify polylactide with zinc oxide nanoparticles (ZnO), raspberry leaf extract (E), and a combined ZnO/extract system (EZnO) in order to prepare novel packaging materials via a solvent-free method, namely cast extrusion. Physicochemical properties: Morphology (GPC, SEM, FTIR), mechanical (tensile tests, puncture), barrier (WVTR, OTR, UV-Vis) and water contact angle for PLA-based films with two thickness ranges were investigated. Additionally, antimicrobial (antibacterial, antifungal and antiviral) tests were performed. GPC results revealed that the presence of the extract counteracted biopolyester degradation during hot melt processing. The best mechanical properties (TS ca. 50 MPa, EB ca. 18%) were obtained for PLA modified with raspberry leaf extract (PLA/E). EZnO addition led to the highest increase in oxygen (with 25%) and water vapor (up to ca. 28%) barrier properties. The material with EZnO addition was also found to be the only one to demonstrate antibacterial effectiveness, although the activity was insignificant. However, the incorporation of EZnO into the biopolymer matrix enhanced its antiviral properties, resulting in the complete inactivation of Φ6 bacteriophage particles used as a surrogate of SARS-CoV-2 virus.

## 1. Introduction

The growing interest in active packaging based on biopolymers is rooted in the need to strive for sustainability—both of the food and of the materials [1,2,3]. The main role of active packaging is the extension of shelf life and prevention of quality loss [3] by using various technologies such as CO_2_ emitters and absorbers, gas (e.g., oxygen, ethylene) scavengers, bioactive (antioxidant, antimicrobial) agents, pouches, sachets, mats, coatings or additives introduced into polymer matrix. There are many advantages to the application of modified packaging, e.g., reducing or completely removing the need to add preservatives into food products. Considering the fact that packaging materials are often disposable, they can be a ballast for the environment. All European Union member countries had to implement the Packaging and Packaging Waste Regulation (PPWR) to reduce packaging waste through sustainable design, utilization of recyclates, or proper labeling [4]. The reduction in environmental pollution by the most conventional plastics, including polyolefins and poly(terephthalate ethylene)—PET, can be reached with biodegradable and bio-based materials. Such polymers can be isolated from natural sources (polysaccharides, i.e., starch, cellulose, alginate; proteins, e.g., zein; polyhydroxyalcanoates isolated from microbial cells) or synthesized from monomers obtained fully or partially from biomass, e.g., polylactide/poly(lactic acid)—PLA, poly(furanoate ethylene)—PEF, or poly(adipate terephthalate ethylene)—PBAT. Thus, these bio-based polyesters can be processed like other thermoplastics and exhibit similar mechanical and barrier properties to oil-based polymers, e.g., polypropylene or PET [5], and new ways to utilize them in food packaging applications are being developed.

PLA can be obtained from lactide by ring opening polymerization or indirect polycondensation of lactic acid [6,7]; thus, there are two names commonly used for PLA: “polylactide” and “poly(lactic acid)”. It is one of the most commercially available bioplastics—some consumers can know it as a “plastic from corn”. It is used not only for food packaging, but can also be applied in other use cases, such as 3D printing [8]. PLA can be obtained from biomass and is fully biodegradable in industrial composting conditions. Its physicochemical properties and tendency to undergo biodegradation depend mainly on the degree of crystallinity. One of the biggest producers of polylactide is NatureWorks LCC [6]. PLA as packaging material in the form of a foil is quite brittle and often needs to be modified in order to be useful in industrial applications. These modifications can be achieved through blending with other biopolyesters, such as PBAT [9], as well as through the addition of plasticizers, many of which are bio-based (e.g., based on esters and fatty acids) to retain the green character of the polymer while also improving elasticity [10,11] and decreasing the sensitivity of PLA to chain scission during thermal processing and making it possible to fine-tune its properties. Antioxidatives and chain extenders can also be introduced to PLA during its processing [12]. Nowadays, traditional and inert food packaging is insufficient for meeting customer requirements, and thus interest in active packaging has rapidly increased. Active packaging refers to materials that improve the quality and extend the shelf life of food products through their functional activity [13], indirectly protecting the consumer’s health. Such packaging may provide high barrier properties against gases and water vapor and can include components such as gas emitters or gas absorbers. In the times of life in a hurry, increasing consumption and convenient food supplied by self-services stores, additional functions such as antimicrobial (including antiviral) properties are new challenges. To modify the polyester, active compounds/agents or fillers are added to confer the required properties [13,14,15,16]. Many active agents can be introduced into the polymer bulk [14] before extrusion, but those that are thermolabile can also be introduced in coating carriers [17,18], via PLA casting from the organic solution [19,20], or through the application of separate elements, such as pads for PLA packaging [21]. These additives can not only functionalize PLA but also affect the thermal and optical properties and alternate polymer chain organization [22]. Essential oils can act as antimicrobial plasticizers, but they tend to decrease barrier properties and transparency [20]. Moreover, they are volatile and can affect the organoleptic properties of stored food. Plant extracts that are rich in, e.g., polyphenols can be used as functional additives for polymers, leading to the creation of completely bio-based materials. Another advantage is their origin; they can be recovered from side products and waste. To boost bioactivity, phenol-rich plant extracts can be hybridized with nanoparticles of metals such as copper, silver or zinc by, e.g., complex formation, or used as reducing agents for the synthesis of nanoparticles and/or as their stabilizers. Among the aforementioned elements, only zinc can be used for packaging which gets into contact with food, as it is considered harmless to human health and has been categorized as GRAS (Generally Recognized as Safe) [23]. Plant extract/ZnO systems can exhibit antibacterial, antifungal, antioxidative and anticancer properties [24,25,26,27]. Additionally, in the work of Alyamani et al. [27], no cytotoxic effect on normal L929 cells had been found. A good source of plant tissue rich in active components are the leaves of different *Rubus* species, e.g., European red raspberry, black raspberry or mountain raspberry. The raspberry leaves are rich in flavan-3-ols, e.g., catechins, flavanols like rutin and kaempferol, and caffeic acid derivatives [28,29]. Thanks to these compounds, the extracts exhibit antimicrobial and antioxidative properties [30,31].

The main goal of the work was to utilize the ethanolic extract (E) of red raspberry leaves—a food processing byproduct—as a functional additive for PLA modification to obtain a fully green material intended for packaging applications. Moreover, the extract was used in a novel way to prepare a hybridized system with nanoZnO, which was supposed to boost the activity of the filler as described in the literature positions mentioned above [23,24,25,26,27]. We also partially relied on our experience from previous work, where ZnO in conjunction with three herbal extracts exhibited synergic antibacterial and antiviral activity, as well as an improvement in the barrier, mechanical and optical properties of PHB/PLA composite films [32]. Here, PLA was extruded with nanoZnO, E, and ZnO in E (EZnO), and foils of two thicknesses were obtained via cast extrusion as an industrial method applicable for large-scale PLA foil production. The influence of the additives, as well as two production processes (regranulation and cast extrusion), on molecular weight was investigated with gel permeation chromatography. Studies of mechanical properties included static elongation and puncture tests. The antiviral and antibacterial properties of the obtained materials were also evaluated to verify if the material could possibly help preserve food products against microbial spoilage or prevent the transmission of diseases spread in transport or by the consumers.

## 2. Results and Discussion

### 2.1. Melt Flow Index (MFI)

Analyzing the data collected in Table 1, it can be seen that the regranulation conditions, including high temperature and shear forces, led to an increase in MFI, from 6.3 for unprocessed PLA to 6.9 for regranulated PLA (rPLA). This could be caused by partial degradation of the polymer during processing, as polylactide is quite susceptible to chain scission (a detailed discussion of this phenomenon is presented in Section 3.2); however, the difference is statistically insignificant. PLA/ZnO exhibited the highest MFI, which can indicate that the filler catalyzed polymer degradation [33]. Interestingly, there is no significant difference between PLA and PLA with raspberry leaf extract, which may possibly be explained by the extract counteracting chain scission. Thus, in a further step of the study, GPC (more unequivocal in the case of changes in molecular weight) of the materials was carried out. Moraczewski et al. [34] reported in their work that the type of extract affected the MFI of PLA. In the case of cinnamon and cocoa extract (0.5 and 1%), MFI of the polyester was not significantly different, whereas MFI of PLA with coffee extract increased noticeably.

### 2.2. GPC Analysis

GPC analysis results for the PLA pellet (first processing) and films obtained via cast extrusion (second processing) are listed in Table 2. It was found that the molecular weight of the bioresin (M_n_ and M_w_) decreased after processing, which confirmed PLA sensitivity to processing conditions, the number of cycles, and the presence of additives causing further degradation [35,36,37]. During hot melt processing, PLA undergoes thermodegradation, hydrolysis and, to a lesser extent, oxidative degradation [35,38]. Comparing regranulated/pelletized PLA, the highest drop was obtained for PLA without additives, caused by shear force and high temperature, as well as for PLA/ZnO (the aforementioned processing conditions + ZnO). The fillers based on zinc compounds led to polymer chain scission, and the higher content of the filler increased the differences in M_n_ and M_w_ [33,39,40]. Due to low ZnO content carried by Atmer, there is no difference in molecular weight after the first processing; it is quite evident that the changes are caused mainly by the extrusion, and the next process caused further chain scission. The lowest drop of the GPC-analyzed parameters can be observed for PLA/E. The polyphenol-rich raspberry leaf extract acted as an antioxidant exhibiting protective activity towards the biopolymer, and this phenomenon is clearly visible after the second processing (extrusion via flat die to obtain tape/film), where M_w_ decreased by 32.9% compared to the original PLA, whereas M_w_ for PLA, PLA/ZnO and PLA/EZnO decreased by 42.8, 44.7 and 46.7%, respectively. The extract might act similarly to synthetic antioxidatives, such as Irganox 1330 or 1010 [36,38]. The greatest drop was obtained for PLA/EZnO film and this can be attributed to the better distribution of ZnO particles in the polymer matrix, leading to a higher degree of degradation catalyzed by the filler. The GPC results correlate with the MFI data.

### 2.3. FTIR Characterization

**Figure 1:**
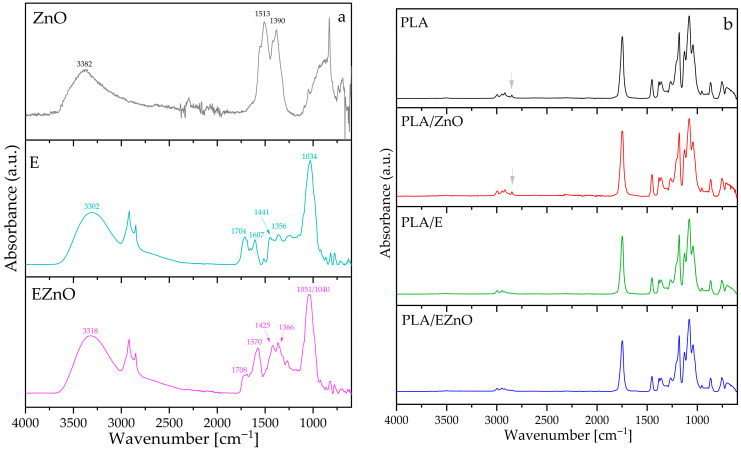
FTIR-ATR spectra of the additives (**a**) and the films (thick): E—raspberry leaf extract, EZnO—the extract with zinc oxide (**b**).

The FTIR-ATR spectra of additives and the films are presented in Figure 1a. It can be seen that E and EZnO were different from each other, indicating effective hybridization with ZnO. In the case of the extract, a similar spectrum was obtained in another work [41] which analyzed raspberry leaf extract obtained through steam explosion. The shift in OH groups from, e.g., hemicellulose with peak at 3302 cm^−1^ for E was shifted to a higher wavenumber 3318 cm^−1^ in EZnO, which can indicate a disruption of hydrogen bonding between plant molecules by ZnO presence. Similar changes at region 1720–800 cm^−1^ were obtained in Stevanović et al.’s work [42] for silver nanoparticles stabilized with raspberry leaf extract. The authors indicated that the intensity of some peaks decreased after interaction with the ions and the functional groups such as NH, (NH)C=0 (from amino acids and proteins). C-O-C and OH played certain role in the complexation and stabilization of nanoparticles by the extract. In our work, it can also be observed that, e.g., the band at 1704 cm^−1^ of C=O group almost disappeared and merged with 1570 cm^−1^ and the peaks in the range of 1720–800 cm^−1^ for EZnO are shifted towards higher wavenumber values, which can be related to zinc complexation with phenolic OH groups [43]. Additionally, the bands at 1513 and 1390 cm^−1^ can probably be assigned to stretching carboxylate groups, which might have originated from the remaining precursors used for nanoparticle formation [44]. These bands have disappeared in the hybridized system, but two bands at 1425 and 1366 cm^−1^ (for aromatic rings and phenolic OH, respectively [45]) became exposed, which can indicate a conjunction between ZnO nanoparticles and the polyphenols from the extract.

Figure 1b shows the FTIR-ATR spectra of the films, confirming PLA as the base polymer in the material [46]. In the wavenumber region 1300–1000 cm^−1^, the stretching C–O bands (1273, 1178, 1077 cm^−1^) can be observed. The ester groups with a high-intensity sharp peak at 1273 cm^−1^, the asymmetric vibrations of O–C at 1077 cm^−1^ and bending of –OH at 1041 cm^−1^ can be found. The peak observed at 865 cm^−1^, as indicated by Siriprom et al. [47], can be attributed to the vibrations of the helical backbone with rocking CH_3_. As can be seen, the presence of the additives did not affect the chemical morphology of the polyester, except for some barely visible changes in the 2990–2840 cm^−1^ region, where the very-low-intensity peak at 2848 cm^−1^ (gray arrow in Figure 1b) disappeared in samples with the extract. Only a few works attempted to find some explanations of the phenomena in this region [17,48]. This band is assigned to a symmetric stretching vibration of the –CH_2_– group [46] that is usually not found in pure PLA and it could come from some additives or impurities in the original Ingeo 4032D. The raspberry leaf extract’s compounds and its dense structure (significant amount of phenolic compounds) can shield methylene groups [17] or these impurities could perhaps degrade during processing. The disappearance of this peak can be found in the work of Amorin et al. [36] for PLA after the fifth cycle of extrusion.

### 2.4. Mechanical Properties

Tensile and puncture test results are presented in Table 3, and it can be noticed that additive presence led to higher tensile strength (TS); in the case of elongation at break (EB), there were no statistically significant differences between unmodified PLA and PLA with additives, except for PLA/E that exhibited the best mechanical properties: the highest values of TS and EB, but also a plasticizing effect (higher EB from 13.3% for unmodified PLA to 17.6%). A slight increase in EB, but with lower TS, was previously obtained in a work which described PLA with hemp extract [49]. The simultaneous increase in both parameters is quite unusual and is worth highlighting, because for many PLA-based materials obtained via extrusion, the introduction of extracts led to an increase in TS with a parallel decrease in EB or decrease in both parameters, e.g., PLA with extracts from *Castanea sativa* Mill. wood [50], wild garlic [51] or artichoke leaves even with the presence of the plasticizer [52]. In the work of Osial et al. [53], EB for PLA with 1% of hydroxyapatite hybridized with curcumin increased to 10% (from 5% for native PLA), with TS remaining essentially the same. A similar trend was observed in the paper by Martins et al. [54] where the EB value for PLA with 1% or 2% green tea extract did not exceed 5%. The lack of flexibility loss is a key advantage of PLA intended for packaging applications, which is generally quite brittle. The introduction of the additives significantly improved puncture resistance and the highest force needed to pierce the foil, with a value of ca. 32% obtained for PLA/EZnO. Due to the fact that tensile strength is calculated based on all three dimensions, thickness affected only the puncture parameter.

### 2.5. Barrier Properties and Contact Angle of PLA

**Figure 2:**
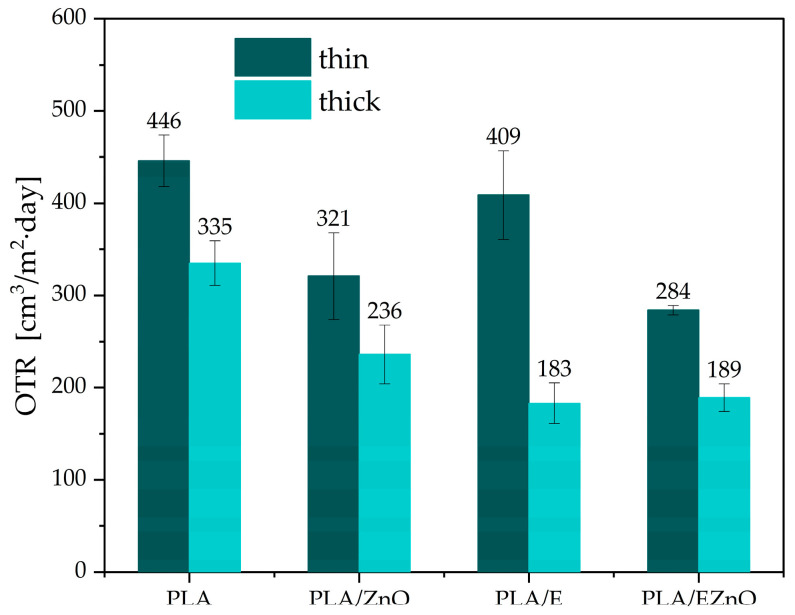
Oxygen transmission rate (OTR) of thin and thick PLA films at 23 °C and RH 50%. E—raspberry leaf extract, EZnO—the extract with zinc oxide.

Transmission rates were determined for oxygen and water vapor (Figure 2, Table 4). Among packaging plastics, PLA exhibits lower O_2_ permeability than polyolefins, namely polyethylene and polypropylene, but higher than PET, and the improvement of barrier properties is a key factor for maintaining the high quality of packed food, i.e., preventing lipid oxidation. In Figure 2, it can be seen that OTR values depended on the type of additive and the thickness of the sample. A higher barrier was obtained in PLA modified with additives containing ZnO. Similar results were obtained for PHB/PLA blends [32]. For ZnO, the decrease in oxygen permeability can be obtained even for a small content of the filler of less than 1% [55,56]. Similarly to OTR, samples with nanofiller exhibited lower permeability (WVTR) than PLA with the extract only. It can be caused by the hydrophobicity of the filler and its solid structure, which creates obstacles in the polymer bulk for transferring gas molecules [57]. It is worth highlighting that the presence of the extract also led to better barrier properties against both gases. This can be caused by the good homogeneity of the polymer.

There is no significant difference in water contact angle values, and WCA for PLA is similar to the value described by Pereira et al. [52]. A slight, but significant, difference can be observed for PLA with the extract, which may be related to the hydrophilic character of the plant additive. The fairly narrow range of the values (ca. 71–75°) can be a result of the low content of additives and their embedding in the polymer bulk.

### 2.6. Optical Properties

**Figure 3:**
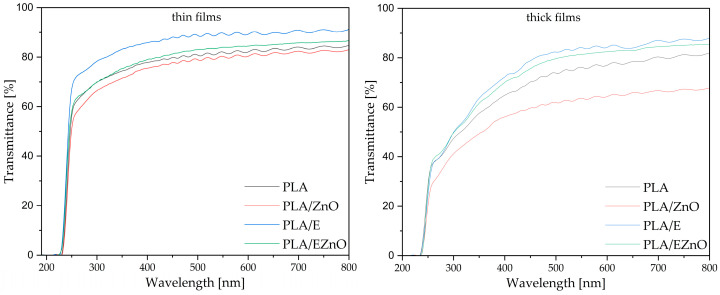
UV-Vis spectra for the films with two ranges of thickness for transmittance mode. E—raspberry leaf extract, EZnO—the extract with zinc oxide.

All the cast-extruded foils are quite transparent, and the highest transprency was obtained for samples containing the extract (PLA/E and PLA/EZnO) (Figure 3). Due to the low content of the additives in the polymer matrix of thin films, their presence did not affect the UV light barrier. It was also found that the thickness of the films affected transparency at the UV-Vis range. For the thick films, UV light transmittance decreased from ca. 400 nm. All the films exibited barrier properties mainly towards the UV-C range of the spectrum. According to the literature, for ZnO, to obtain UV-barrier properties of PLA, there is a need for a higher content of the filler [33,58]. Similarly, in the case of plant extracts in the PLA matrix [59], their addition absorbs UV light, but parallely, leads to lower transparency in visual light. The highest transparency (91%) was obtained for thin PLA/E (Table 5).

The results of color measurements for PLA thin foils are presented in Table 5 as values of CIELab three-dimensional color space. L* indicates lightness (black at 0, white at 100), values on the a* axis indicate colors from red (negative) to green (positive), while values on the b* axis indicate colors from blue to yellow. All samples were characterized by highest lightness, without significant changes after the additive introduction. The addition of ZnO did not affect the color of PLA. Due to the intensively colored extract, green coloration (attributed to chlorophyll) can be observed in PLA with E. The green tint of PLA/E is more bright, and EZnO caused green pigmentation with a slightly yellow/beige tint (higher—a* shift towards red thus can be registered as a brownish tint), which is also reflected as a higher YI value; thus, the former can be more visually attractive.

### 2.7. Morphology—Scanning Electron Microscopy (SEM)

**Figure 4:**
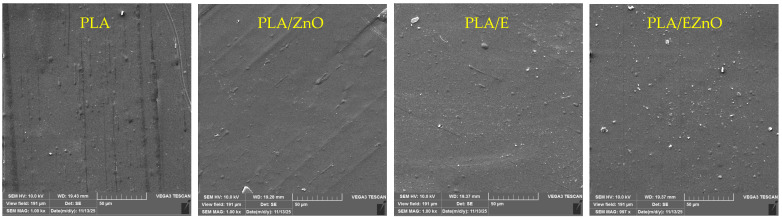
SEM micrograph of PLA and modified PLA surface. EZnO—the extract with zinc oxide 1000× magnitude).

All films were smooth when observed with the naked eye, but they are also smooth in microscale, homogenous, and without defects like scratches, roughness, pores or microcracks (Figure 4). On SEM micrographs of PLA/ZnO, there are no visible zinc oxide aggregates or impurities. Slight grittiness can be noticed in PLA/E, but it does not affect its visual appearance (see Figure 4). Slightly different observations were reported by Pereira et al. [53], who incorporated artichoke leaf extract into a PLA matrix. The authors noted non-uniform dispersion of the extract, with visible agglomerates within the PLA matrix, which was likely due to insufficient interfacial adhesion between the extract particles and the polymer. Some small aggregates (0.4–0.5 µm) were found in PLA/EZnO, but they were well-dispersed in the polymer matrix. The aggregation can also occur for higher content of ZnO nanofiller [60]. It can indicate that raspberry leaf extract can induce ZnO aggregation or small clusters formation (similarly, e.g., as cynnamaldehyde [20]), in opposite to mixed herbal extract from celandines, nettle and St. John’s wort, which led to better dispersion of nanoZnO [32]. Good dispersion of the additives in the PLA matrix is correlated with the improvement of mechanical (Table 3) and barrier properties (Table 4, Figure 2) of the film.

### 2.8. Antimicrobial Properties Analysis

The results of microbiological analysis showed that PLA films with ZnO nanoparticles were not effective against either *S. aureus*, *E. coli* or *Botrytis cinerea*, as they neither inhibited bacterial and mold growth nor reduced the number of viable cells (Table 6).

Comparable findings were reported in a previous study [32], in which zinc oxide nanoparticles were incorporated into the PHB/PLA matrix. On the other hand, PLA modified with ZnO nanoparticles [61] has been reported to exhibit slight antibacterial activity against *S. aureus* (with a reduction in bacterial count of less than 2 log) while remaining ineffective against *E. coli*. However, in that study the concentration of nanoparticles in the biopolymer matrix (0.14 pph) was higher than the one used in the present work (0.0375 pph). The PLA film containing raspberry leaf extract incorporated into the matrix exhibited slight activity against *S. aureus* (Table 6), while no effectiveness against *E. coli* was observed. The PLA with the combined additive EZnO was found to be inactive against both *S. aureus* and *E. coli* strains. The results showed that no synergistic effect between nanoZnO and the extract was observable, differing from the previous work [32] where a polyester blend with ZnO hybridized with mixed herbal extracts was effective against *S. aureus* and *E. coli*. Dai et al. [62] reported that PLA/ZnO significantly reduced the growth rate of *S. aureus*, indicating strong antibacterial activity against this microorganism. However, this effect decreased over time. The authors also found that incorporating pomegranate peel extract (PPE) into PLA imparted antibacterial properties against *S. aureus*, which was attributed to the gradual release of PPE from the polymer matrix. Furthermore, the antibacterial performance of the composite film was markedly improved when ZnO nanoparticles were combined with PLA modified with PPE, confirming a synergistic effect between these active additives. Another authors’ study [63] also demonstrated a synergistic effect between active agents incorporated into polymeric coating. However, in that case, supercritical CO_2_ extracts from raspberry seeds, pomegranate seeds, and rosemary were used as the active substances. Pereira et al. [52] incorporated an artichoke leaf extract into PLA matrix active films which could possess antibacterial properties. The authors noted that the obtained materials presented 0.21–1.15 log reduction against *E. coli* and 0.65–0.93 log reduction against *S. aureus*, depending on the extract concentration in the matrix. These results confirmed that modified PLA was slightly effective towards the mentioned strains, showing a trend similar to the observations made during our study. He et al. [64] prepared PBAT/PLA/*Rosmarinus officinalis* L. extract blend films with the addition of 0.1, 0.3, and 0.5% of the extract using melt extrusion, followed by blown film processing. The authors analyzed the antibacterial properties of the film against *S. aureus* and *E. coli* cells. For both bacterial strains, the activity increased with increasing extract concentration. The authors suggested that the observed concentration-dependent antibacterial activity could be attributed, among other factors, to improvements in the interfacial structure of the blends, which may have hindered bacterial adhesion and proliferation, as well as to the release of active compounds from the biopolymer matrix. They proposed that the released substances could interfere with bacterial metabolism or DNA replication. Considering these findings, it may be assumed that the lack of antibacterial activity observed for PLA films containing raspberry leaf extract in the present study could be due to the insufficient release of active compounds from the polymer matrix or due to an insufficient extract concentration. These assumptions are further supported by the lack of antifungal activity observed for the modified PLA films. As shown in Table 6, neither the films containing raspberry leaf extract nor those incorporating both the plant extract and ZnO nanoparticles exhibited activity against *Botrytis cinerea*, even though Kucharski et al. [65] reported that raspberry leaf extract was effective against mold. Several studies have demonstrated that PLA-based blends incorporating plant-derived compounds exhibit antifungal activity. For instance, PLA films containing thymol and carvone effectively inhibited the growth of fungal pathogens [66]. Moreover, PLA materials modified with plant extracts such as rosemary have been reported to exhibit antifungal activity due to the presence of terpene compounds. These findings suggest that the incorporation of plant extracts rich in terpenes and essential oils such as rosemary into PLA/PBAT matrices may confer antifungal properties, supporting their potential application [67]. The drawbacks of these additives are their lower thermal stability and their fragrance, which could affect packed food, whereas polyphenolic compounds and nanoparticles are odorless.

**Figure 5:**
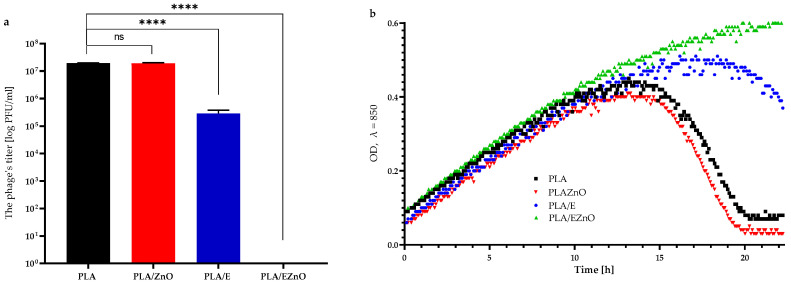
(**a**) The influence of the films on the Φ6 phage (Φ6) titer. One-way ANOVA; ****—*p* < −0.0001; ns—*p* > 0.5). (**b**) The optical density (OD) over time of *P. syringe* with Φ6 particles after its cultivation with unmodified (PLA) and modified films. PLA/ZnO—PLA film with ZnO; PLA/E—PLA film with raspberry leaf extract; PLA/EZnO—PLA film with raspberry leaf extract and ZnO nanoparticles.

The results of the antiviral analysis showed a decrease in optical density (OD) after 15 h of incubation of *P. syringae* with Φ6 particles that had been previously exposed to the control samples (PLA and PLA/ZnO), indicating that these films were not effective against the viral particles (Figure 5). Moreover, the titers of the phage lysates obtained for PLA and PLA/ZnO samples differed only slightly. For the PLA/E film, a reduction in OD was observed after 21 h of incubation of *P. syringae* with the Φ6 bacteriophage previously cultured in the presence of this film (Figure 5b). In addition, the titer of the Φ6 lysate for the PLA/E sample was significantly lower compared to the control (Figure 5b). These findings suggest that the film containing red raspberry leaf extract exhibited moderate antiviral activity against this bacteriophage. Similar observations were reported in the previous study [32], where the incorporation of plant extracts into a biopolymer matrix did not significantly enhance antiviral performance. In contrast, other results [68] showed no decrease in OD for *P. syringae* cultures inoculated with Φ6 lysate previously incubated with polymer films containing scCO_2_ extracts from raspberry seeds, pomegranate seeds, and rosemary, which meant that the viral particles were successfully inactivated. In the case of the PLA/EZnO film, no decrease in OD was detected even after 23 h of incubation of *P. syringae* with Φ6 phage propagated in the presence of this material. These findings differ from those reported in our previous work [32]. Overall, the results indicate that PLA films modified with both raspberry leaf extract and ZnO nanoparticles exhibited strong antiviral activity. A synergistic effect between the plant extract and ZnO nanoparticles was also observed. While the PLA/E film showed moderate effectiveness against the Φ6 bacteriophage—an enveloped virus often used as a surrogate for SARS-CoV-2—the PLA/EZnO film led to complete inactivation of the phage particles. Based on these findings, it may be suggested that PLA/EZnO film, being effective against Φ6, could also be active towards SARS-CoV-2 particles. This assumption is supported by the work of Serrano-Aroca [69], who investigated both Φ6 and SARS-CoV-2 and validated this biosafe viral model using a wide range of materials. The materials analyzed by the author were shown to inactivate a very high percentage (from 94.92 to 100%) of both Φ6 and SARS-CoV-2 particles after comparable contact times. The author concluded that the Φ6 bacteriophage can serve as a reliable and safe surrogate for SARS-CoV-2 in antiviral studies involving various biomaterials, composites, nanomaterials, nanocomposites, coatings, extracts, and chemical compounds.

## 3. Materials and Methods

### 3.1. Materials

Polylactide (PLA) Ingeo^TM^ 4032D (NatureWorks, Minnetonka, MN, USA) was used as the polymer matrix. Zinc oxide (ZnO) nanoparticles were supplied by Permedia Colors (Lublin, Poland), and Atmer110^TM^ (bio-based additive based on ethoxylated sorbitan ester) by CRODA (now Cargill Bioindustrial, Gouda, The Netherlands) was used as the carrier for ZnO and EZnO. Dried raspberry leaves (Zakład Konfekcjonowania Ziół Flos Elżbieta i Jan Głąb, Wieluń, Poland) were purchased in a local store; 98% ethanol, used for the extraction, was purchased from Warchem (Warsaw, Poland). The examination of antibacterial effectiveness was performed using selected bacteria: *Staphylococcus aureus* DSM 346 and *Escherichia coli* DSM 498 strains. *Botrytis cinerea* DSM 5144 was used to examine antifungal effectiveness. *Pseudomonas syringae* van Hall 1902 DSM 21482 was used as the host for the Φ6 bacteriophage (a SARS-CoV2 surrogate). The Φ6 bacteriophage DSM-21518 was selected to examine antiviral activity of the films. The bacterial strains and Φ6 phage were purchased from the Leibniz Institute Deutsche Sammlung von Mikroorganismen und Zellkulturen (DSMZ, Braunschweig, Germany). To analyze the antimicrobial properties of the films, MacConkey agar, Tryptic Soy Agar (TSA), Lysogeny Broth (LB), Tryptic Soy Broth (TSB), potato broth and potato agar from BioMaxima (Lublin, Poland) were used. All media were suspended in 1 L of distilled water and sterilized in a Prestige Medical “Classic” autoclave at 121 °C for 15 min (Płock, Poland).

### 3.2. Methods

#### 3.2.1. Preparation of the Additives

Dried raspberry leaves (100 g) were added to a 1000 mL bottle topped up with 98% ethanol. Then, the bottle was sealed, microwaved (Amica, Wronki, Poland) for 15 min at 70 °C, transferred to a shaker (IKA, Staufen im Breisgau, Germany) and agitated for 1 h at 70 °C (150 rpm). The extraction procedure was based on the method previously reported in our work [32], with minor modifications. The ethanolic extract was subsequently separated from the leaves using a Büchner funnel. The filtrate was divided into two fractions: one for pure extract, and the other for subsequent hybridization nanoZnO. For both fractions, 10 g of Atmer was introduced to obtain E and EZnO additive. To the last one, 0.3 g of ZnO nanoparticles was added. The two systems were left for the solvent evaporation (at 55 °C) to obtain dark green-colored final additives.

#### 3.2.2. Preparation of PLA-Based Films

The films were prepared as follows: In the first step, the neat, dried (for 4 h at 70 °C) biopolyester pellet was mixed by hand with the prepared additives, namely E (Atmer 1.25 pph and the evaporated extract 0.2 pph per 100 pph of PLA) and EZnO (Atmer, extract + 0.0375 pph ZnO), and then blended in a co-rotating twin-screw extruder (L/D = 40) at a temperature profile of 160/170 × 9 °C at 40 rpm and pelletized. Then, the extrudate of neat and modifed PLA were cast-extruded (L/D = 30) with a flat die (width of the film ca. 17 cm) using the following temperature profile: 170/175/180/180 °C, co-extruder 180 °C, die 180 °C, 60 rpm. For the processing, LabTech line (Labtech Engineering Co., Ltd., Samut Prakan, Thailand) was applied.

#### 3.2.3. Physicochemical Characteristics of the Extrudate

Melt flow index (MFI) of the original PLA and the extrudates was determined at 190 °C with 2.16 kg weight using RB-M plastometer (Rolbatch Gmbh, Germany) according to the ASTM D 1238 standard [70].

For the Gel Permeation Chromatography (GPC) analysis of the extrudate (pellet) and thick films, samples were dissolved in HPLC-grade, ethanol-stabilized chloroform (Scharlab S.L, Barcelona, Spain). They were then analyzed on a KNAUER Smartline HPLC (KNAUER Wissenschaftliche Geräte GmbH, Berlin, Germany) with a tandem column set consisting of PFG Lux Linear XL (PSS Polymer Standards Service GmbH, Mainz, Germany) and StyDiViBe-P-10E7A-BPT (AppliChrom GmbH, Oranienburg, Germany) columns thermostated at 30 °C, with UV detection set for 240 nm and the same HPLC-grade chloroform used as the eluent, with a constant flow rate of 1 ml/min.

Chemical morphology was characterized through a FTIR spectrophotometric analysis using a Perkin Elmer spectrophotometer (Spectrum 100, Waltham, MA, USA) equipped with ATR. The samples were analyzed with 16 scans in 4000–650 cm^−1^ range with SPECTRUM software (version 10.03.06.0100).

Mechanical tests performed using Zwick//Roell Z2.5 machine (Ulm, Germany) included measurements of tensile strength [32] and puncture of thin (41–56 µm) and thick (115–140 µm) films. Puncture resistance was measured according to the ASTM F1306 standard [71] with some modifications: the films were cut into 12 mm-wide strips and the testing speed of the needle was 100 mm/min. At least eight replications for each series were performed, and the parameters with standard deviations were calculated using the TestExpert v1.6 software. Statistical analysis was subjected to one-way ANOVA, and the statistical significance was determined using a *t*-Student test.

The barrier properties investigation was conducted for two gases: oxygen and water vapor. The oxygen transmission rate—OTR—for the films was determined according to ASTM D3985 standard [72] using the OX-TRAN Model 2/10 (Mocon, Minneapolis, MN, USA) at a temperature of 23 °C and a relative humidity (RH) of 50%. Water vapor transmission rate was determined using procedure based on ASTM F1249-20 standard [73] with PERMATRAN 3/33 (Mocon, Minneapolis, MN, USA) at RH 100% with compensation at 90% at 37 ± 2 °C.

Water contact angle determination at room temperature (RT) was performed with SEO contact analyzer Phoenix-Mini (PM-041807, Suwon, Republic of Korea) using Surfaceware 8 software to measure the value after distilled water drop deposition.

UV-Vis characterization was carried out with a Thermo Scientific Evolution 220 spectrophotometer (Waltham, MA, USA). Transparency was measured as transmittance at ʎ = 700 nm, and UV-Vis absorption capacity was evaluated in the wavelength range of 190–900 nm.

The colorimeter CR-5 (Konica Minolta, Tokyo, Japan) was applied to measure color changes in CIELab color scale. Samples were analyzed in 5 repetitions at random locations on each sample. Color changes (∆E*), yellowness index (YI) and chroma (*c) were calculated according to the equations given in work [74].

The surface morphology of the films was analyzed using scanning electron microscopy (SEM). Initially, the samples were mounted on pin stubs and coated with a thin gold layer using a sputter coater at 24 °C (Quorum Technologies Q150R S, Brighton and Hove, East Sussex, UK). Subsequently, SEM images were recorded with a Vega 3 LMU microscope (Tescan, Brno-Kohoutovice, Czech Republic). The observations were performed using a tungsten filament at an accelerating voltage of 10 kV.

#### 3.2.4. Evaluation of the Antimicrobial Properties

The films were cut into 30 × 30 mm squares. The antibacterial and antifungal properties of PLA, PLA/ZnO, PLA/E, and PLA/EZnO films were then evaluated in accordance with the ASTM E 2180-01 standard [75], following procedures described previously by the authors [32,62,63,76] with slight modification. The incubation time of plates containing mold (*Botrytis cinerea*) increased to 48 h. The incubation temperature was 23 °C. For the antiviral assessment, Φ6 phage lysate was purified using the method reported by Bhetwal et al. [77], and subsequently prepared according to the procedure described by Bonilla et al. [78]. The antiviral performance of the modified films was compared with that of neat PLA (control samples) and determined based on a modified ISO 22196:2011 protocol [79]. Finally, amplification of Φ6 bacteriophage particles was conducted following the approach proposed by Skaradzińska et al. [80]. To monitor the host’s (*Pseudomonas syringae*) growth in real time, after its contact with the films (PLA, PLA/ZnO, PLA/E, and PLA/EZnO), Φ6 lysate was incubated separately with each film according to the ISO 22196:2011 standard [79], with minor modifications as previously described by the authors [68,76,81]. The LB was placed in BioSan bioreactors (BS-010160-A04, BioSan, Riga, Latvia). Then, an overnight culture of *P. syringae* was inoculated into 20 mL of LB and incubated at 28 °C until an optical density (OD) of 0.2 was reached. A total of four Φ6 lysates were amplified in *P. syringae* cultures: one after incubation with neat PLA (control), and three after incubation with PLA/E, PLA/ZnO, and PLA/EZnO films, respectively. In the final stage, 100 µL of Φ6 phage lysate (MOI = 1) was added to the bacterial culture (OD = 0.2), followed by incubation for 24 h at 28 °C.

## 4. Conclusions

In our study, we successfully modified PLA to obtain a packaging material with additional functionality. ZnO, raspberry leaf extract and a hybrid system consisting of both additives were used as modifiers. PLA films were obtained via a solvent-free casting method by extrusion in two types of thickness. The results of GPC analysis revealed that the introduction of E reduced chain scission during hot melt processing, whereas additives with ZnO acted oppositely. The presence of the extract significantly improved the mechanical properties, and the elongation at break values (25% higher for PLA/E than PLA) indicate that the extract exhibited plasticizing activity without loss in tensile strength; moreover, for PLA/E, the highest transparency was obtained. This modification caused only slight antibacterial activity against *S. aureus* (reduction of 98%), which was likely due to the low concentration of the extract in the matrix. Hybridization of ZnO with the extract led to the best barrier properties towards oxygen and water vapor. The improvement in mechanical and barrier properties is related to good dispersion of the modifier in the polyester matrix. This, in addition to the synergistic effect between both active agents, could have been the reason for the high antiviral activity of PLA/EZnO film.

To summarize, raspberry leaf extract acted as a multifunctional additive: plasticizer, colorant, protective agent (antioxidant) against degradation during hot melt processing, and antiviral agent. The antiviral properties of food packaging materials could be important in the post-pandemic era due to limiting the spread of viruses via consumers’ hands.

Modified PLA materials with improved physicochemical properties, functionalized by additional antimicrobial activity and characterized by attractive visual appearance, can be used not only as safe and fully bio-based active packaging which meet the standards for green chemistry and food production, but also have the potential for other applications.

The presented work on the materials will be developed further, with specific focus on their thermal and antimicrobial properties, the influence of material modification on their characteristics, as well as their performance during food storage tests.

## Figures and Tables

**Table 1:** MFI values for PLA extrudates at 190 °C with 2.16 kg weight. E—raspberry leaf extract, EZnO—the extract with zinc oxide.

Sample	MFI [g/10 min]
PLA original	6.28 ± 0.57 ^b^
rPLA	6.88 ± 0.66 ^b^
rPLA/ZnO	8.37 ± 0.84 ^a^
rPLA/E	6.30 ± 0.74 ^b^
rPLA/EZnO	6.78 ± 0.58 ^b^

^a,b^—Averages marked with the same letters do not differ significantly from each other for *p* < 0.05.

**Table 2:** GPC results for original PLA (before processing), PLA extrudate (in twin-screw extruder) and films obtained by cast extrusion. PDI—polydyspersity index; E—raspberry leaf extract, EZnO—the extract with zinc oxide.

Sample	M_n_ [g/mol]	M_w_ [g/mol]	PDI
pellet			
PLA original	50,950	120,540	1.98
rPLA	35,630	77,520	2.19
rPLA/ZnO	32,060	78,010	2.45
rPLA/E	40,390	89,970	2.27
rPLA/EZnO	34,730	82,050	3.36
foil (thick)			
PLA	31,980	68,900	2.15
PLA/ZnO	29,330	66,620	2.29
PLA/E	39,110	80,860	2.08
PLA/EZnO	27,960	64,200	2.29

**Table 3:** Mechanical properties (from tensile and puncture test). I—length of deformation at pierce; E—raspberry leaf extract, EZnO—the extract with zinc oxide.

Sample	Static Elongation			Puncture	
	YM [MPa]	TS [MPa]	EB [%]	Force [N]	I [mm]
thin					
PLA	1682 ± 275 ^a^	37.6 ± 3.0 ^c^	13.3 ± 0.9 ^b^	4.19 ± 0.21 ^c^	1.39 ± 0.29 ^b^
PLA/ZnO	1684 ± 418 ^a^	43.3 ± 4.2 ^b^	15.0 ± 1.4 ^b^	5.01 ± 0.55 ^b^	2.17 ± 0.29 ^a^
PLA/E	2063 ± 349 ^a^	51.1 ± 3.9 ^a^	17.6 ± 2.5 ^a^	4.81 ± 0.63 ^b^	1.30 ± 0.25 ^b,c^
PLA/EZnO	1775 ± 274 ^a^	44.0 ± 4.8 ^b^	14.0 ± 2.0 ^b^	6.13 ± 0.43 ^a^	1.03 ± 0.24 ^c^
thick					
PLA	1551 ± 301 ^a^	42.8 ± 5.1 ^a^	14.2 ± 3.6 ^a,b^	7.97 ± 0.54 ^b^	0.87 ± 0.14 ^b^
PLA/ZnO	1594 ± 303 ^a^	39.2 ± 8.0 ^b^	14.0 ± 2.6 ^b^	9.67 ± 1.16 ^a^	2.25 ± 0.24 ^a^
PLA/E	1749 ± 446 ^a^	47.8 ± 3.6 ^a^	16.3 ± 1.4 ^a^	9.21 ± 0.99 ^a^	0.92 ± 0.28 ^b^
PLA/EZnO	1594 ± 475 ^a^	44.6 ± 2.1 ^a,b^	15.3 ± 0.9 ^a,b^	9.46 ± 1.04 ^a^	0.58 ± 0.20 ^c^

^a–c^—averages marked with the same letters do not differ significantly from each other for *p* < 0.05.

**Table 4:** Water vapor transmission rate (WVTR) at 37 °C and water contact angle (WCA) at ambient conditions of PLA films. E—raspberry leaf extract, EZnO—the extract with zinc oxide.

Sample	WVTR _RH100%_ [g^2^/m^2^∙Day]	WVTR _RH90%_ * [g/m^2^∙Day]	WCA [°]
thin			
PLA	222 ± 14	200 ± 13	74.0 ± 0.4
PLA/ZnO	191 ± 2	172 ± 2	74.6 ± 3.7
PLA/E	196 ± 14	180 ± 11	72.7 ± 1.0
PLA/EZnO	165 ± 15	145 ± 13	73.1 ± 1.8
thick			
PLA	109 ± 2	87 ± 2	74.9 ± 2.5
PLA/ZnO	78 ± 5	71 ± 3	75.2 ± 1.6
PLA/E	87 ± 3	79 ± 1	71.5 ± 2.5
PLA/EZnO	81 ± 1	72 ± 1	70.8 ± 2.2

* compensated.

**Table 5:** Results of color measurement in CIELab scale and transpatency T (transmittance at 700 nm) for thin films. E—raspberry leaf extract, EZnO—the extract with zinc oxide.

Sample	CIELab Scale						T _700 nm_ [%]
	L*	a*	b*	ΔE	YI	C*	
PLA	97.7 ± 0.07	0.05 ± 0.01	0.05 ± 0.01	Ref.	0.29	0.21	84
PLA/ZnO	97.6 ± 0.02	0.05 ± 0.01	0.05 ± 0.03	0.02	0.44	0.30	82
PLA/E	97.0 ± 0.09	−0.62 ± 0.09	−0.62 ± 0.21	2.76	2.28	1.67	91
PLA/EZnO	96.8 ± 0.11	−0.78 ± 0.26	−0.78 ± 0.95	8.95	4.32	3.03	86

**Table 6:** The influence of the films on *S. aureus* and *E. coli*; PLA—PLA film; PHA/ZnO—PLA film with ZnO in the biopolymer matrix; PLA/E—PLA film with a raspberry leaf extract; PLA/EZnO—PLA film with a raspberry leaves extract and ZnO nanoparticles.

Sample	*S. aureus*	*E. coli*	*B. cinerea*
% reduction			
PLA	0	0	0
PLA/ZnO	88	42	44
PLA/E	98	39	7
PLA/EZnO	89	66	81

## Data Availability

The original contributions presented in this study are included in the article. Further inquiries can be directed to the corresponding author.

## Abbreviations

The following abbreviations are used in this manuscript:

E	Red Raspberry Leaf Extract
EB	Elongation at Break
FTIR	Fourier Transform Infrared Spectroscopy
GPC	Gel Permeation Chromatography
OD	Optical Density
OTR	Oxygen Transmission Rate
PLA	Polylactide
SEM	Scanning Electron Microscopy
TS	Tensile Strength
UV-Vis	Ultraviolet–Visible Light Spectroscopy
WCA	Water Contact Angle
WVTR	Water Vapor Transmission Rate
ZnO	Zinc Oxide

## References

[1] Alves J., Gaspar P.D., Lima T.M., Silva P.D. (2023). What Is the Role of Active Packaging in the Future of Food Sustainability? A Systematic Review. J. Sci. Food Agric..

[2] López-Miguens M.J., Álvarez-González P., Dopico-Parada A. (2025). Can Active and Intelligent Packaging Support Sustainability in Food Sector? Insights from a Consumer’s Viewpoint. Waste Manag..

[3] Chrysochou P., Tiganis A. (2025). Active, Intelligent or Sustainable? A Comparative Study of Consumer Preferences for Food Packaging Technologies. Packag. Technol. Sci..

[4] https://environment.ec.europa.eu/topics/waste-and-recycling/packaging-waste_en.

[5] Zdanowicz M., Paszkiewicz S., El Fray M. (2025). Polyesters and Deep Eutectic Solvents: From Synthesis through Modification to Depolymerization. Prog. Polym. Sci..

[6] Aniśko J., Barczewski M. (2021). Polylactide: From Synthesis and Modification to Final Properties. Adv. Sci. Technol. Res. J..

[7] Yu J., Xu S., Liu B., Wang H., Qiao F., Ren X., Wei Q. (2023). PLA Bioplastic Production: From Monomer to the Polymer. Eur. Polym. J..

[8] Andrzejewski J., Das S., Lipik V., Mohanty A.K., Misra M., You X., Tan L.P., Chang B.P. (2024). The Development of Poly(Lactic Acid) (PLA)-Based Blends and Modification Strategies: Methods of Improving Key Properties towards Technical Applications—Review. Materials.

[9] Ostrowska J., Sadurski W., Paluch M., Dębowski M., Wrona O., Sołtan K., Tyński P. (2024). PLA/PBAT Blends for Blown Film Extrusion. Polimery.

[10] Mena-Prado I., Fernández-García M., Blázquez-Blázquez E., Muñoz-Bonilla A., del Campo A. (2025). Plasticizing PLA with Biobased Fatty Esters: Comprehensive Study on Film Properties. J. Polym. Environ..

[11] Sun S., Weng Y., Zhang C. (2024). Recent Advancements in Bio-Based Plasticizers for Polylactic Acid (PLA): A Review. Polym. Test..

[12] Sun H., Luo W., Weng Y., Zhang C. (2025). Advances in Poly(Lactic Acid) Chain Extenders: Mechanisms, Performance, and Sustainability. J. Vinyl Addit. Technol..

[13] Zende R., Ghase V., Jamdar V. (2025). Recent Advances in the Antimicrobial and Antioxidant Capabilities of PLA Based Active Food Packaging. Polym.-Plast. Technol. Mater..

[14] Dejene B.K. (2025). Reviewing the Manufacturing Challenges and Scientific Debates: Insights into the Antibacterial Capabilities and Potential Applications of PLA/ZnO Nanocomposites. J. Thermoplast. Compos. Mater..

[15] Li J., Sun H., Weng Y. (2024). Natural Extracts and Their Applications in Polymer-Based Active Packaging: A Review. Polymers.

[16] Velásquez E., Patiño Vidal C., Rojas A., Guarda A., Galotto M.J., López de Dicastillo C. (2021). Natural Antimicrobials and Antioxidants Added to Polylactic Acid Packaging Films. Part I: Polymer Processing Techniques. Compr. Rev. Food Sci. Food Saf..

[17] Mizielińska M., Zdanowicz M., Tarnowiecka-Kuca A., Bartkowiak A. (2024). The Influence of Functional Composite Coatings on the Properties of Polyester Films before and after Accelerated UV Aging. Materials.

[18] Fiorentini C., Leni G., de Apodaca E.D., Fernández-de-Castro L., Rocchetti G., Cortimiglia C., Spigno G., Bassani A. (2024). Development of Coated PLA Films Containing a Commercial Olive Leaf Extract for the Food Packaging Sector. Antioxidants.

[19] Olewnik-Kruszkowska E., Gierszewska M., Wrona M., Richert A., Rudawska A. (2023). Polylactide-Based Films Incorporated with Berberine—Physicochemical and Antibacterial Properties. Foods.

[20] Qin Y., Liu D., Wu Y., Yuan M., Li L., Yang J. (2015). Effect of PLA/PCL/Cinnamaldehyde Antimicrobial Packaging on Physicochemical and Microbial Quality of Button Mushroom (*Agaricus bisporus*). Postharvest Biol. Technol..

[21] Vieira T.M., Alves V.D., Moldão Martins M. (2022). Application of an Eco-Friendly Antifungal Active Package to Extend the Shelf Life of Fresh Red Raspberry (*Rubus idaeus* L. Cv. ‘Kweli’). Foods.

[22] Rojas A., Velásquez E., Vidal C.P., Guarda A., Galotto M.J., de Dicastillo C.L. (2021). Active PLA packaging films: Effect of processing and the addition of natural antimicrobials and antioxidants on physical properties, release kinetics, and compostability. Antioxidants.

[23] Espitia P.J.P., Otoni C.G., Soares N.F.F. (2025). Zinc Oxide Nanoparticles for Food Packaging Applications. Antimicrobial Food Packaging.

[24] Elumalai K., Velmurugan S. (2015). Green synthesis, characterization and antimicrobial activities of zinc oxide nanoparticles from the leaf extract of *Azadirachta indica* (L.). Appl. Surf. Sci..

[25] Sharmila G., Thirumarimurugan M., Muthukumaran C. (2019). Green synthesis of ZnO nanoparticles using Tecoma castanifolia leaf extract: Characterization and evaluation of its antioxidant, bactericidal and anticancer activities. Microchem. J..

[26] Ramesh M., Anbuvannan M., Viruthagiri G. (2015). Green synthesis of ZnO nanoparticles using Solanum nigrum leaf extract and their antibacterial activity. Spectrochim. Acta-Part A Mol. Biomol. Spectr..

[27] Alyamani A.A., Albukhaty S., Aloufi S., Almalki F.A., Al-Karagoly H., Sulaiman G.M. (2021). Green fabrication of zinc oxide nanoparticles using phlomis leaf extract: Characterization and in vitro evaluation of cytotoxicity and antibacterial properties. Molecules.

[28] Pavlović A.V., Papetti A., Zagorac D.Č.D., Gašić U.M., Mišić D.M., Tešić Ž.L., Natić M.M. (2016). Phenolics Composition of Leaf Extracts of Raspberry and Blackberry Cultivars Grown in Serbia. Ind. Crops Prod..

[29] Oszmiański J., Wojdyło A., Nowicka P., Teleszko M., Cebulak T., Wolanin M. (2015). Determination of Phenolic Compounds and Antioxidant Activity in Leaves from *Wild rubus* L. Species. Molecules.

[30] Zielonka-Brzezicka J., Nowak A., Zielińska2 M., Klimowicz A. (2016). Comparison of the Antioxidant Properties of Selected Parts of Raspberry (*Rubus idaeus*) and Blackberry (*Rubus fruticosus*). Pomeranian J. Life Sci..

[31] Staszowska-Karkut M., Materska M. (2020). Phenolic Composition, Mineral Content, and Beneficial Bioactivities of Leaf Extracts from Black Currant (*Ribes nigrum* L.), Raspberry (*Rubus idaeus*), and Aronia (*Aronia melanocarpa*). Nutrients.

[32] Zdanowicz M., Mizielińska M., Kowalczyk A. (2024). Cast Extruded Films Based on Polyhydroxyalkanoate/Poly(Lactic Acid) Blend with Herbal Extracts Hybridized with Zinc Oxide. Polymers.

[33] Shojaeiarani J., Bajwa D., Jiang L., Liaw J., Hartman K. (2019). Insight on the Influence of Nano Zinc Oxide on the Thermal, Dynamic Mechanical, and Flow Characteristics of Poly(Lactic Acid)–Zinc Oxide Composites. Polym. Eng. Sci..

[34] Moraczewski K., Stepczyńska M., Malinowski R., Karasiewicz T., Jagodziński B., Rytlewski P. (2019). The effect of accelerated aging on polylactide containing plant extracts. Polymers.

[35] Velghe I., Buffel B., Vandeginste V., Thielemans W., Desplentere F. (2023). Review on the Degradation of Poly(Lactic Acid) during Melt Processing. Polymers.

[36] Amorin N.S.Q.S., Rosa G., Alves J.F., Gonçalves S.P.C., Franchetti S.M.M., Fechine G.J.M. (2014). Study of Thermodegradation and Thermostabilization of Poly(Lactide Acid) Using Subsequent Extrusion Cycles. J. Appl. Polym. Sci..

[37] Mysiukiewicz O., Barczewski M., Skórczewska K., Matykiewicz D. (2020). Correlation between Processing Parameters and Degradation of Different Polylactide Grades during Twin-Screw Extrusion. Polymers.

[38] Hallstein J., Metzsch-Zilligen E., Pfaendner R. (2024). Long-Term Thermal Stabilization of Poly(Lactic Acid). Materials.

[39] Pantani R., Gorrasi G., Vigliotta G., Murariu M., Dubois P. (2013). PLA-ZnO Nanocomposite Films: Water Vapor Barrier Properties and Specific End-Use Characteristics. Eur. Polym. J..

[40] Murariu M., Benali S., Paint Y., Dechief A.-L., Murariu O., Raquez J.-M., Dubois P. (2021). Adding Value in Production of Multifunctional Polylactide (PLA)–ZnO Nanocomposite Films through Alternative Manufacturing Methods. Molecules.

[41] Zheng X., Yang J., Zhao Y., Zhou X., Zhang N., Liu Y. (2025). Efficient extraction, characterization, and systematic evaluation of bound polyphenols in raspberry leaf residues. Biomass Convers. Biorefinery.

[42] Stevanović M.S., Zvezdanović J.B., Stanojević L.P., Stanojević J.S., Petrović S.M., Cakić M.D., Cvetković D.J. (2019). Synthesis, characterization and antioxidant activity of silver nanoparticles stabilized by aqueous extracts of wild blackberry (*Rubus* spp.) and raspberry (*Rubus idaeus* L.) leaves. Adv. Technol..

[43] Wang X., Feng Y., Chen C., Yang H., Yang X. (2020). Preparation, characterization and activity of tea polyphenols-zinc complex. LWT.

[44] Huang G.G., Wang C.-T., Tang H.-T., Huang Y.-S., Yang J. (2006). ZnO Nanoparticle-Modified Infrared Internal Reflection Elements for Selective Detection of Volatile Organic Compounds. Anal. Chem..

[45] Si M., Yan X., Liu M., Shi M., Wang Z., Wang S., Zhang J., Gao C., Chai L., Shi Y. (2018). In Situ Lignin Bioconversion Promotes Complete Carbohydrate Conversion of Rice Straw by *Cupriavidus basilensis* B-8. ACS Sustain. Chem. Eng..

[46] Grabowska B., Kaczmarska K., Cukrowicz S., Mączka E., Bobrowski A. (2020). Polylactide Used as Filment in 3D Printing–Part 1: FTIR, DRIFT and TG-DTG Studies. J. Cast. Mater. Eng..

[47] Siriprom W., Sangwaranatee N., Chantarasunthon K., Teanchai K., Chamchoi N. (2018). Characterization and Analyzation of the Poly (L-Lactic Acid) (PLA) Films. Mater. Today Proc..

[48] Valerini D., Tammaro L., Villani F., Rizzo A., Caputo I., Paolella G., Vigliotta G. (2020). Antibacterial Al-Doped ZnO Coatings on PLA Films. J. Mater. Sci..

[49] Tutek K., Rosiak A., Kałużna-Czaplińska J., Masek A. (2024). Biodegradable PLA-Based Materials Modified with Hemp Extract. Polym. Test..

[50] Lombardi A., Dominici F., Campo M., Vignolini P., Fochetti A., Pizzetti M., Papalini M., Luzi F., Bernini R., Puglia D. (2026). Effects of Phenolic-Rich Extracts from Castanea Sativa Mill. Wood Processing Byproducts on the Development of Compostable Polylactic Acid-Based Materials with Antioxidant and Antibacterial Properties. ACS Omega.

[51] Radusin T., Tomšik A., Šarić L., Ristić I., Giacinti Baschetti M., Minelli M., Novaković A. (2019). Hybrid Pla/Wild Garlic Antimicrobial Composite Films for Food Packaging Application. Polym. Compos..

[52] Pereira P.F.M., Qazanfarzadeh Z., Sowinski P., Jiménez-Quero A. (2026). Bioactive PLA Packaging Films Using Artichoke Leaf Extract as Functional Additive. Food Packag. Shelf Life.

[53] Osial M., Wilczewski S., Godlewska U., Skórczewska K., Hilus J., Szulc J., Roszkiewicz A., Dąbrowska A., Moazzami Goudarzi Z., Lewandowski K. (2024). Incorporation of Nanostructural Hydroxyapatite and Curcumin Extract from *Curcuma longa* L. Rhizome into Polylactide to Obtain Green Composite. Polymers.

[54] Martins C., Vilarinho F., Sanches Silva A., Andrade M., Machado A.V., Castilho M.C., Sá A., Cunha A., Vaz M.F., Ramos F. (2018). Active Polylactic Acid Film Incorporated with Green Tea Extract: Development, Characterization and Effectiveness. Ind. Crops Prod..

[55] Tang Z., Fan F., Chu Z., Fan C., Qin Y. (2020). Barrier Properties and Characterizations of Poly(Lactic Acid)/ZnO Nanocomposites. Molecules.

[56] Nasab M.S., Tabari M. (2018). Antimicrobial Properties and Permeability of Poly Lactic Acid Nanocomposite Films Containing Zinc Oxide. Nanomed. Res. J..

[57] Marano S., Laudadio E., Minnelli C., Stipa P. (2022). Tailoring the Barrier Properties of PLA: A State-of-the-Art Review for Food Packaging Applications. Polymers.

[58] Lizundia E., Penayo M.C., Guinault A., Vilas J.L., Domenek S. (2019). Impact of ZnO Nanoparticle Morphology on Relaxation and Transport Properties of PLA Nanocomposites. Polym. Test..

[59] Nansu W., Chaiwut P., Ross S., Ross G., Suphrom N., Mahasaranon S. (2021). Developments of Biodegradable Polymer Based on Polylactic Acid (PLA) with Natural Color Extracts for Packaging Film Applications. J. Met. Mater. Miner..

[60] Shankar S., Wang L.F., Rhim J.W. (2018). Incorporation of Zinc Oxide Nanoparticles Improved the Mechanical, Water Vapor Barrier, UV-Light Barrier, and Antibacterial Properties of PLA-Based Nanocomposite Films. Mater. Sci. Eng. C.

[61] Mizielińska M., Kowalska U., Jarosz M., Sumińska P., Landercy N., Duquesne E. (2018). The Effect of UV Aging on Antimicrobial and Mechanical Properties of PLA Films with Incorporated Zinc Oxide Nanoparticles. Int. J. Environ. Res. Public Health.

[62] Dai L., Li R., Liang Y., Liu Y., Zhang W., Shi S. (2022). Development of Pomegranate Peel Extract and Nano ZnO Co-Reinforced Polylactic Acid Film for Active Food Packaging. Membranes.

[63] Ordon M., Nawrotek P., Stachurska X., Mizielińska M. (2021). Polyethylene Films Coated with Antibacterial and Antiviral Layers Based on CO_2_ Extracts of Raspberry Seeds, of Pomegranate Seeds and of Rosemary. Coatings.

[64] He X., Tang L., Huang R. (2026). Active Packaging Films from PBAT/PLA with *Rosmarinus officinalis* L. Extract: Antioxidant, UV-Shielding, and Compostable Properties. Molecules.

[65] Kucharski Ł., Cybulska K., Kucharska E., Nowak A., Pełech R., Klimowicz A. (2022). Biologically Active Preparations from the Leaves of Wild Plant Species of the Genus Rubus. Molecules.

[66] Jawaid M., Boonruang K., Chinsirikul W., Hararak B., Kerddonfag N., Chonhenchob V., Kenawy E.-R. (2016). Antifungal Poly(lactic acid) Films Containing Thymol and Carvone. MATEC Web Conf..

[67] Klinmalai P., Srisa A., Laorenza Y., Katekhong W., Harnkarnsujarit N. (2021). Antifungal and plasticization effects of carvacrol in biodegradable poly(lactic acid) and poly(butylene adipate terephthalate) blend films for bakery packaging. LWT.

[68] Ordon M., Zdanowicz M., Nawrotek P., Stachurska X., Mizielińska M. (2021). Polyethylene Films Containing Plant Extracts in the Polymer Matrix as Antibacterial and Antiviral Materials. Int. J. Mol. Sci..

[69] Serrano-Aroca Á. (2022). Antiviral Characterization of Advanced Materials: Use of Bacteriophage Phi 6 as Surrogate of Enveloped Viruses Such as SARS-CoV-2. Int. J. Mol. Sci..

[70] (2023). Standard Test Method for Melt Flow Rates of Thermoplastics by Extrusion Plastometer.

[71] (2025). Standard Test Method for Slow Rate Penetration Resistance of Flexible Barrier Films and Laminates.

[72] (2017). Standard Test Method for Oxygen Gas Transmission Rate Through Plastic Film and Sheeting Using a Coulometric Sensor.

[73] (2017). Standard Test Method for Water Vapor Transmission Rate Through Plastic Film and Sheeting Using a Modulated Infrared Sensor.

[74] Viñas-Ospino A., Anticona M., Esteve M.J., Blesa J., Mizielińska M., Zdanowicz M. (2025). Poly(Butylene Succinate) Films Modified with Functional Deep Eutectic Solvent/Orange Peel Extract Systems. Food Packag. Shelf Life.

[75] (2002). Standard Test Method for Determining the Activity of Incorporated Antimicrobial Agent(s) in Polymeric or Hydrophobic Materials.

[76] Jankowski W., Mizielińska D., Mizielińska M. (2025). Influence of Q-SUN Irradiation on Antimicrobial and Antiviral Activity of Tea Tree Oil-Based Coatings on Polypropylene Films. Appl. Sci..

[77] Bhetwal A., Maharjan A., Shakya S., Satyal D., Ghimire S., Khanal P.R., Parajuli N.P. (2017). Isolation of Potential Phages against Multidrug-Resistant Bacterial Isolates: Promising Agents in the Rivers of Kathmandu, Nepal. Biomed. Res. Int..

[78] Bonilla N., Rojas M.I., Netto Flores Cruz G., Hung S.-H., Rohwer F., Barr J.J. (2016). Phage on tap–a quick and efficient protocol for the preparation of bacteriophage laboratory stocks. PeerJ.

[79] (2011). Measurement of Antibacterial Activity on Plastics and Other Non-Porous Surfaces.

[80] Skaradzińska A., Ochocka M., Śliwka P., Kuźmińska-Bajor M., Skaradziński G., Friese A., Roschanski N., Murugaiyan J., Roesler U. (2020). Bacteriophage Amplification—A Comparison of Selected Methods. J. Virol. Methods.

[81] Zdanowicz M., Barczewski M., Mizielińska M., Miądlicki P. (2025). Physico-Chemical, Rheological, and Antiviral Properties of Poly(Butylene Succinate) Biocomposites with Terpene—Hydrophobized Montmorillonite. Polymers.

